# As to the Efficacy of Coca Preparations

**Published:** 1887-01

**Authors:** H. Liebermann

**Affiliations:** Paris


### *AS TO THE EFFICACY OF COCA PREPARATIONS.*

I desire to state, for the benefit of my colleagues, the results which I have obtained during my long career as military surgeon by the use of *Vin Coca Mariani.* Briefly stated, I have used it with the greatest success in profound senœmia, resulting from long, arduous campaigns in tropical countries, and in the gastro-intestinal irritation with loss of appetite and dyspepsia, which is such a frequent accompaniment of this condition. Two or three wine-glasses of *Vin Mariani* each day relieved the debility with wonderful rapidity, inasmuch as the tolerance of the stomach for nourishing food and the appetite were restored by its administration. Mariani's wine is vastly superior to the wine of quinia, since the latter, by augmenting the gastro-intestinal irritation, interferes with alimentation, and consequently with repair, thereby aggravating the anæmia instead of ameliorating it.

I have also employed it in those cases of chronic alcoholism, fortunately rare in the French army, which follow the abuse of absinthe and strong liquors. Mariani's wine, while producing primarily a certain amount of cerebral stimulation, exercised a predominant sedative effect upon the nervous system. I have, moreover, witnessed the spectacle of hardened drunkards giving up their pernicious habits and returning to a normal condition under the influence of this treatment.

I have also employed Mariani's wine successfully in the treatment of the tobacco habit. A few glasses of the wine, taken in small swallows or mixed with water, were sufficient to replace both pipes and cigars, since the patients obtained the cerebral stimulation which they sought for, albeit unconsciously.

I have also employed it in chronic bronchitis, and even pulmonary phthisis. Mariani's wine augments the appetite and diminishes the cough in both these conditions. When combating the cough, I have given it mixed with water—a wine-glass of the wine to a tumbler of spring water.

Finally, I have employed it in the convalescence following typhoid fever with the greatest success, and this in cases where the irritability of the stomach was so great that no wine, not even Bordeaux, could be tolerated.

To recapitulate: I am convinced that Mariani's wine is the most potent arm which can be placed in the hands of the military surgeon for the purpose of combating the sickness, infirmities and vicious habits engendered by campaigning and the hardships of military life. I will also state that when any other than Mariani's preparations ot Coca were used, the results intended were not produced; quite the contrary: bad effects, and even unpleasant complications, were noticeable, and to this I call the special attention of the physician.—*H. Liebermann, M. D., Paris.*

